# gga-miR-146c Activates TLR6/MyD88/NF-κB Pathway through Targeting MMP16 to Prevent *Mycoplasma Gallisepticum* (HS Strain) Infection in Chickens

**DOI:** 10.3390/cells8050501

**Published:** 2019-05-24

**Authors:** Kang Zhang, Yun Han, Zaiwei Wang, Yabo Zhao, Yali Fu, Xiuli Peng

**Affiliations:** Key Laboratory of Agricultural Animal Genetics, Breeding and Reproduction Ministry of Education, College of Animal science and Technology and College of Veterinary Medicine, Huazhong Agricultural University, Wuhan 430070, China; zhangkang123@webmail.hzau.edu.cn (K.Z.); hany@webmail.hzau.edu.cn (Y.H.); wangzaiwei@webmail.hzau.edu.cn (Z.W.); zyb@webmail.hzau.edu.cn (Y.Z.); FYL@webmail.hzau.edu.cn (Y.F.)

**Keywords:** *Mycoplasma gallisepticum*, chicken, miR-146c, MMP16, NF-κB signaling pathway

## Abstract

*Mycoplasma gallisepticum* (*MG*), a pathogen that infects chickens and some other birds, triggers chronic respiratory disease (CRD) in chickens, which is characterized by inflammation. The investigation of microbial pathogenesis would contribute to the deep understanding of infection control. Since microribonucleic acids (miRNAs) play a key role in this process, gga-mir-146c, an upregulated miRNA upon *MG* infection, was selected according to our previous RNA-sequencing data. In this paper, we predicted and validated that *MMP16* is one of gga-miR-146c target genes. Results show that *MMP16* is the target of gga-miR-146c and gga-miR-146c can downregulate *MMP16* expression within limits. gga-miR-146c upregulation significantly increased the expression of TLR6, NF-κB p65, MyD88, and TNF-α, whereas the gga-miR-146c inhibitor led to an opposite result. gga-miR-146c upregulation effectively decreased apoptosis and stimulated DF-1 cells proliferation upon *MG* infection. On the contrary, gga-miR-146c inhibitor promoted apoptosis and repressed the proliferation. Collectively, our results suggest that gga-miR-146c upregulation upon MG infection represses *MMP16* expression, activating TLR6/MyD88/NF-κB pathway, promoting cell proliferation by inhibiting cell apoptosis, and, finally, enhancing cell cycle progression to defend against host *MG* infection.

## 1. Introduction

*Mycoplasma*, a prokaryotic parasite, has negative impacts on human health and livestock farming [1,2,3]. Of those, *Mycoplasma gallisepticum* (*MG*) could cause severe chronic respiratory disease (CRD) in chickens and sinusitis in turkeys, represented by severely inflamed tracheas, lungs, and air sacs [4,5]. Controlling the impact of the disease on a global level is done by vaccination and medication. Unfortunately, they cannot eliminate all pathogens of infected chickens, and some vaccines may revert to virulence [6,7,8]. *MG* infection results in considerable losses consisting of reducied weight gain and egg production, as well as increased embryo morbidity [9].

Microribonucleic acids (miRNAs) act as critical regulators of gene expression by binding to the 3′-untranslated regions (3′-UTRs) with sequence complementarity and decreasing the stability or translation efficiency of target mRNAs [10]. Increasing evidence suggests that miRNAs can extensively regulate various physiological and pathological processes and serve as important regulators of the defense and inflammatory responses to the host, including pathogenesis of many avian diseases [11,12]. Recent researches have indicated this. For instance, miR-181 and miR-29c might act as a Marek’s disease tumor suppressor by targeting MYBL1 and inhibitor of accelerated avian influenza virus replication, respectively [13,14]. gga-miR-375 may act as a critical role in avian leucosis tumorigenesis [15], while gga-miR-2127 attenuated antiviral innate immune response by targeting bursal disease virus [16]. Our previous reports found that gga-miR-19a, gga-miR-99a, and gga-miR-101-3p play an important role in *MG*-HS (*Mycoplasma gallisepticum* HS strain) infection [17,18,19].

NF-κB signaling not only regulates cell proliferation and apoptosis, but also relates to inflammatory response upon TLR activation [20]. NF-κB is normally maintained inactively in the cytoplasm by binding with a member of the inhibitory kappa B (IκB) family. Upon proinflammatory stimulation, it could be phosphorylated and proteolytically degraded to promote nuclear NF-κB to translocate and combine with target genes, which function in various biological processes [21]. Many miRNAs were indicated to take part in the regulation of the NF-κB signaling pathway at multiple steps [22].

The miR-146 family consists of miR-146a, miR-146b, and miR-146c. MiR-146a plays pivotal roles in regulating the proliferation of immune cells and inhibiting NF-κB dependent inflammatory responses [23,24]. Moreover, miR-146a can be sustained expressed by stimulation of TLR2 [25]. MiR-146b might regulate bacteria recognition and the inflammatory response in Mycobacterium avium subspecies paratuberculosis infection [26]. The upregulation of miR-146b was found to be closely associated with the pathogenesis of pulmonary artery remodeling in ascites syndrome in broiler chickens. In addition, activation of TLR4 signaling could upregulate miR-146b expression in human monocytes. [27,28]. The miR-146c, potentially targeting immune response-related genes, is upregulated in other types of influenza-infected chicken cells or tissues [29], and in tumorous spleens and lymphomas infected with Marek’s disease virus [30]. Current knowledge demonstrates that miR-146 family can prevent the development of harmful inflammatory responses.

Our previous miRNAs deep sequencing results revealed gga-miR-146c was significantly upregulated in embryonic lungs of chickens upon *MG* infection [31], suggesting that gga-miR-146c might be functional in response to MG-HS infection. It was validated in this study that gga-miR-146c is remarkably upregulated in embryonic lungs of chickens and DF-1 cell lines with *MG* infection. gga-miR-146c was functional by regulating TLR6/MyD88/NF-κB pathway and targeting *MMP16* to manipulate cell cycle, multiplication, and apoptosis in host defense of *MG*-HS infection.

## 2. Materials and Methods

### 2.1. Ethics Declaration

The treatment program on chicken embryo had been approved by Use Committee of Huazhong Agricultural University and the Institutional Animal Care. These procedures were operated in accordance with the approved rules.

### 2.2. Mycoplasma Strains

*MG*-HS (Mycoplasma gallisepticum HS strain) was separated from a chicken farm in Hubei province, China [32,33]. *MG*-HS culture and its concentration determination were described in the previous article [32], measured by a color-changing unit (CCU) assay [34].

### 2.3. Cell infection Experiments

DF-1 cells were purchased from Huiying (Shanghai, China). Trypsin treatment was used to detach the DF-1 cells from cell culture, evenly inoculated into six-well plates, and then cultured in the antibiotics free medium. All experiments were repeated three independent times. The DF-1 cells were infected with *MG* (1 × 10^10^ CCU/mL, 100 μL) when the cell density was about 50%–60%. After 48 h infection, we used Trizol (Invitrogen, Carlsbad, CA, USA) to collect cells for further experiments.

### 2.4. gga-miR-146c Target Gene Prediction

To forecast the potential gga-miR-146c targets, TargetScan (v7.2, Whitehead Institute for Biomedical Research, Cambridge, MA, USA, http://www.targetscan.org/) and miRDB (Washington University, St. Louis, MO, USA, http://www.mirdb.org/miRDB/) were used. The conservation of target genes was analyzed according to TargetScan. The mFE between gga-miR-146c and its seed sequence 3′-UTR was from RNA hybrid (Bielefeld University, Bielefeld, Germany, http://bibiserv.techfak.uni-bielefeld.de/rnahybrid/). The analysis of genes functions was based on DAVID Bioinformatics Resources (v6.8, Laboratory of Human Retrovirology and Immunoinformatics, Frederick, MD, USA, http://david.abcc.ncifcrf.gov/).

### 2.5. RNA Oligonucleotides and DNA Primers

The primers are included in Table S1. Table S2 lists the sequences of RNA oligonucleotides. gga-miR-146c mimics (marked as miR-146c) and inhibitor (marked as miR-146c-Inh) were designed by GenePharma (Shanghai, China). There was a random miRNA mimic (marked as miR-146c-NC) and a random miRNA inhibitor (marked as miR-146c-Inh-NC) that were not found to suppress any chicken target genes, and they were served as the negative controls.

### 2.6. Dual-Luciferase Reporter Assay

In order to construct the reporter plasmid, MMP16 3′-UTR covering the seed sequence binding site was amplified by RT-PCR. The cDNA template was extracted from chicken embryo lung tissues, extracting the normal luciferase reporter plasmid, then mutating three core sequences through PCR. The amplified products included the sites of the enzyme cut *Xho* I/*Not* I. The primer sequences were showed in Table S1.

DF-1 cells were seeded on 24-well plates, and 2 × 10^5^ cells per well were used for the luciferase assay. Next, 10 pmol miR-146c, miR-146c-NC, and 200 ng reporter plasmid were transfected into cells together by Lipofectamine 3000 (Invitrogen). The cells were gathered to measure dual-luciferase activity 48 h transfection later by using Lumat LB 9507 Ultra Sensitive Tube Luminometer (Titertek Berthold, Nanjing, China). The luciferase activity of each sample was normalized by the firefly luciferase activity and the Renilla luciferase activity. All experiments were carried out three independent repeats.

### 2.7. RNA Extraction and RT-qPCR

The total RNA was extracted with Trizol Reagent (Invitrogen). The purification of RNA was carried out using RNeasy microcolumns on the basis of the manufacturer’s protocol (Qiagen, Valencia, CA, USA). TransStart Top Green qPCR SuperMix (TRANSGEN, Beijing, China) was used to mix with total RNA, then each RNA sample was tested by RT-qPCR on CFX96 or CFX384 TouchTM (Bio-Rad, Hercules, CA, USA). The relative expression of gga-miR-146c, TLR6, MMP16, NF-κB p65, MyD88, and TNF-α were calculated by Ct (2^−ΔΔCt^) method. To analyze the data, 7500 software v.2.0.1 (Applied Biosystems, Foster City, CA, USA) was used. The internal control of gga-miR-146c was 5S-RNA, and the internal control of TLR6, MMP16, NF-κB p65, TNF-α, and MyD88 was glyceraldehyde-3-phosphate dehydrogenase (GAPDH). Table S1 showed the primers. The experiment was carried out three independent times.

There were 6 × 10^3^ DF-1 cells and 100 μL DMEM containing 10% (*v*/*v*) FBS in each well on six-well plates. Then plates were putted into an incubator overnight (39 °C, 5% CO_2_). When the cells grew to the right density, 7.5 pmol of miR-146c, miR-146c-NC, miR-146c-Inh, or miR-146c-Inh-NC and Lipofectamine 3000 were added to the six-well plates. In addition, the blank control was a mock transfection (marked as blank). The cells were collected after transfection of 48 h. Total RNA was extracted and purified, TransStart Top Green qPCR SuperMix (TRANSGEN) was used to mix with total RNA, then each RNA sample was tested by RT-qPCR on the CFX96 or CFX384 TouchTM (Bio-Rad). There were three independent transfections. We used the 2^−ΔΔCt^ method to calculate the relative mRNA expression of TLR6, NF-κB p65, MyD88, and TNF-α [35].

### 2.8. Immunofluorescence

Luciferase assay was performed on 24-well plates with 2 × 10^5^ DF-1 cells in per well. Lipofectamine 3000 (Invitrogen) was adopted to transfect the 10pmol of miR-146c, miR-146c-NC, miR-146c-Inh, miR-146c-Inh-NC, and 200 ng reporter plasmid into1 cells. Then, cells were fixed by 4% paraformaldehyde for 15 min at 48 h post-transfection. Nonspecific binding sites were blocked by incubating in 1% BSA for 30 min. Then incubate sections for 60 min with anti-rabbit NF-κB p65 antibody (diluted at 1:100). After another wash in PBS, NF-κB p65 antibody was incubated with goat anti-rabbit FITC-labeled IgG (1:1000, Bioswamp, Wuhan, China) at 37 ◦C for an hour. After washed, the cells were counterstained using DAPI. The stained macrophages were magnified 200× *g*.

### 2.9. ELISA

There were 6 × 10^3^ DF-1 cells and 100 μL DMEM in each well on six-well plates. Then, plates were placed into an incubator overnight (39 °C, 5% CO_2_). Before transfection, phosphate-buffered saline (PBS) was used to wash DF-1 cells twice, and Opti-MEMI ReduMced Serum was added to the plates. When the cells grew to the right density, 7.5 pmol of miR-146c, miR-146c-NC, miR-146c-Inh, or miR-146c-Inh-NC and Lipofectamine 3000 were added to the six-well plates. In addition, the blank control was a mock transfection (marked as the blank group). The cells were gathered after 48 h transfection. Then, cell culture supernates were collected in the six-well plates and centrifugation at 3000× *g*, 4 °C, for 10 min. The MyD88, TLR6, and TNF-α were tested by ELISA kit instruction (YUANYE, Shanghai, China).

### 2.10. Western-Blot Analysis

When cells plated in 24-well plates grew to the right density, indicated RNA oligonucleotides were transfected into cells. After transfection of 48 h, RIPA-buffer (Beyotime, Beijing, China) with 100 mM phenylmethanesulfonyl fluoride (PMSF) was used to isolate the total proteins from the DF-1 cells. Bicinchoninic acid (BCA) protein assay reagent kit (Beyotime) was used to test the Protein concentrations. Then, total protein was separated with 12% sodium dodecylsulfate-polyacrylamide gel electrophoresis (SDS-PAGE). Protein was transferred to polyvinylidene fluoride (PVDF) membranes (Beyotime) for 2 h at 80 mA. Five percent (*w*/*v*) fat-free milk was used to block the membranes at normal atmospheric temperature for 1 h. Next, anti-β-actin served as a loading control, and the membrane was incubated with goat polyclonal anti-MMP16 (Sigma-Aldrich, St. Louis, MO, USA) and rabbit anti-β-actin overnight at 4 °C. The membrane was incubated with rabbit anti-goat secondary antibody for 1 h after TBST washing. The enhanced chemiluminescence (ECL) detection system (Bio-Rad) was used to detect antigen-antibody complexes on the membranes. All the tests were triplicated.

### 2.11. Cell Proliferation, Cycle, and Apoptosis

DF-1 cells were seeded in six-well plates at 6 × 10^3^ cell/well 100 μL of DMEM containing 10% (*v*/*v*) FBS, then they were placed into an incubator overnight at 39 °C with 5% CO2. Then, the miR-146c, miR-146c-NC, miR-146c-Inh, or miR-146c-Inh-NC was transfected into the DF-1 cells as described above. When the cells grew to the right density, cells were infected by *MG*-HS (7 μL, 10^10^ CCU/mL). Ten microliter CCK-8 solution was each added 24 h, 48 h, and 72 h after transfection, and incubated at 39 °C for 4 h. Uninfected *MG* cells (blank *MG*-) and the infected *MG* cells (miR-free *MG*+) were regarded as controls. The Cell Counting Kit-8 (CCK-8, DOJINDO, Shanghai, China) was adopted to test cell proliferation. The optical density of each well plate at 450 nm was measured by microplate reader (Bio-Rad).

Transfection treatments were described above. Cells were infected by *MG*-HS (7 μL, 10^10^ CCU/mL) after transfection for 4 h. After transfection for 48 h, 70% ethanol was used to immobilize cells. MiR-free-*MG*+ and blank *MG*- served as controls. Cell cycle detection kit (KeyGEN, Nanjing, China) was used to analyze cell cycle and flow cytometer calculated percentages of cells in G1, S, and G2 phases. Annexin V, FITC apoptosis detection kit (DOJINDO) was used to test the cell apoptosis. Percentages of cells in four quadrants were calculated. All the tests were triplicated.

### 2.12. Statistical Analysis

All experiments were carried out three times. Data were mean ± SD and performed using Student’s *t*-test. * *p* < 0.05, ** *p* < 0.01 mean significant differences.

## 3. Results

### 3.1. gga-miR-146c Expression Was Upregulated after MG Infection

**Figure 1:**
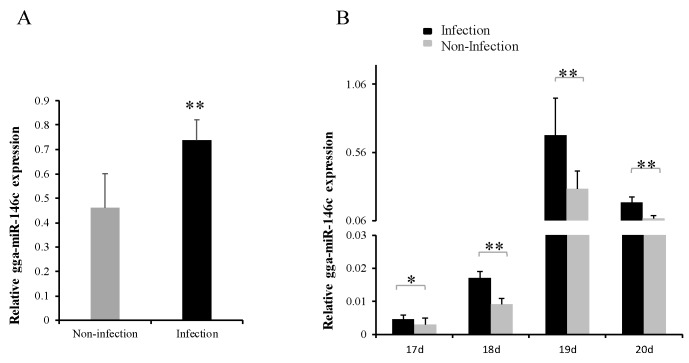
*Mycoplasma gallisepticum* (MG) infection generated gga-miR-146c upregulation. Relative gga-miR-146c expression in MG noninfected or infected DF-1 cells (**A**) and in 17 days to 20 days (8 to 11 days post-infection) chicken embryo lung tissues (**B**). RT-qPCR was performed to assess gga-miR-146c expression that normalized to 5S-rRNA. Uninfected groups served as negative controls. Values are mean ± SD, * *p* < 0.05, ** *p* < 0.01.

The qRT-PCR results showed gga-miR-146c expression was significantly upregulated in *MG*-infected DF-1 cells comparing with noninfected ones (Figure 1A). Subsequently, we further identified gga-miR-146c expression in chicken embryos upon *MG*-HS infection on the ninth day of hatching (total 21 days of egg hatching). Eight to eleven days after infection (amount to 17 to 20 days of egg hatching), gga-miR-146c expression was remarkably upregulated in *MG*-infected embryonic chicken lungs comparing with its expression in the noninfected lungs of chicken embryos (Figure 1B).

### 3.2. gga-miR-146c Contains Conserved Target Site of MMP16

**Figure 2:**
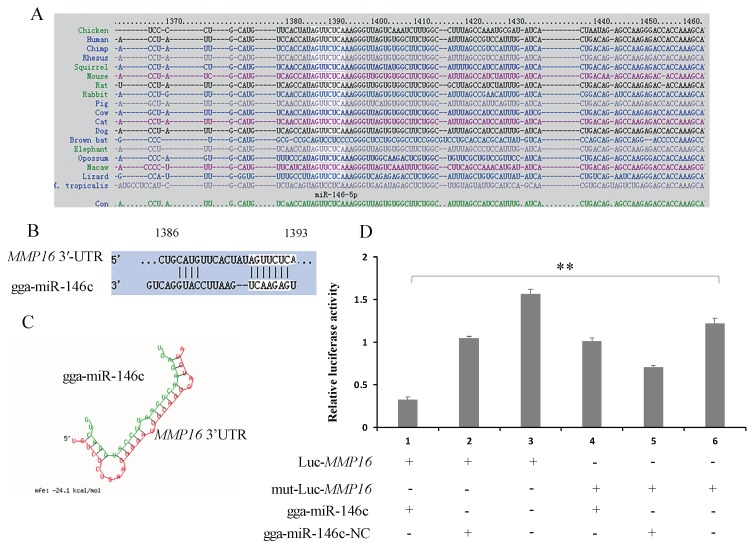
*MMP16* is a target of gga-miR-146c. (**A**) Alignment of MMP16 3′-UTR from different species. The highlighted sequence is conserved region; (**B**) Alignments of gga-miR-146c and the target site in MMP16 3′-UTR. The highlighted part is gga-miR146c seed sequence; (**C**) Structure of gga-miR-146c and MMP16 3′-UTR target site. Red strand represents target sequence and green represents gga-miR146c; (**D**) Luc-MMP16 (3′-UTR) and either gga-miR-146c or gga-miR-146c-NC were co-transfected into cells. The dual-luciferase glow assay was performed 24 h transfection later. Data from three experiment results were mean ± SD. Two-tailed Student’s *t*-test, ** *p* < 0.01.

With a combined use of online software (miRDB, miRbase, and TargetScan) to analysis gga-miR-146c, *MMP16* was selected as a possible target of gga-miR-146c due to a high prediction score (score 94). Target site sequence in the MMP-16 3′-UTR was conserved across human, mouse, rat, pig, etc. (Figure 2A). The predicted target site is located at 1371–1393 bps, and the seed site is between 1386—1392 bps (Figure 2B). The minimum free energy (mFE) between MMP16 3′-UTR and gga-miR-146c is about −24.1 kCal/mol, which indicates high stability (Figure 2C).

### 3.3. MMP16 Is a Direct Target Gene of gga-miR-146c

To validate the direct binding of gga-miR-146c to 3′-UTR of *MMP16* gene, a luciferase reporter gene analysis (psi-CHECK™-2) was performed. The luciferase reporter vector Luc-MMP16 containing a potential seed sequence of MMP16 3′-UTR was constructed and co-transfected with gga-miR-146c mimics (miR-146c) for 48 h. MMP16 3′-UTR luciferase activity was effectively decreased with the application of gga-miR-146c mimics, while no effect on luciferase activity was observed in negative control (nontargeting) (miR-146c-NC) (Figure 2D). To determine that interaction between miR-146c with the 3′-UTR of MMP16 caused the reduction of luciferase activity, at the same time, we co-transfected miR-146c mimics and a mutant dual luciferase reporter that contained three mutations in the seed region into DF-1 cells. As expected, no remarkable effect was observed when applied with miR-146c mimics (Figure 2D).

**Figure 3:**
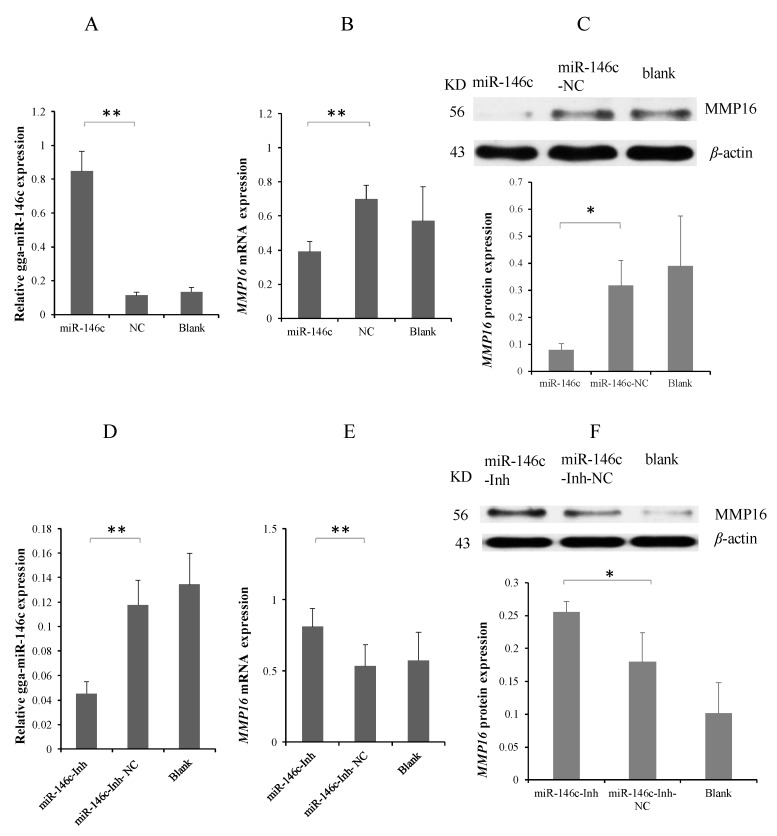
gga-miR-146c regulates MMP-16 expression. (**A**) The detection of gga-miR-146c overexpression; (**B** and **C**) DF-1 cells were transfected with gga-miR-146c mimics. MMP16 messenger RNA (mRNA) and protein expression was respectively measured by reverse transcription polymerase chain reaction (RT-qPCR) or Western blot, normalized to glyceraldehyde 3-phosphate dehydrogenase (GAPDH) orβ-actin; (**D**) gga-miR-146c expression level was reduced with inhibitor transfection; (**E**,**F**) MMP16 mRNA and protein expression with gga-miR-146c inhibitor transfection was detected by RT-qPCR or Western blot respectively, normalized to GAPDH or β-actin; Values are mean ± SD, * *p* < 0.05, ** *p* < 0.01.

To explore the interaction of gga-miR-146c and MMP16 3′-UTR, miR-146c mimics, miR-146c-NC, and miR-146c-Inh or miR-146c-Inh-NC were transfected into the DF-1 cells to validate the expression of endogenous MMP16 using Western blot and qRT-PCR. gga-miR-146c was overexpressed by mimic transfection (Figure 3A). Overexpression of gga-miR-146c resulted in marked reduction of MMP16 (Figure 3B,C). On contrary, the miR-146c inhibitor inhibited the expression of gga-miR-146c markedly (Figure 3D), with led to the upregulation of MMP16 (Figure 3E,F). These data show that *MMP16* is a direct target gene of gga-miR-146c, and negatively regulated by binding to MMP16 3′-UTR in DF-1 cells.

### 3.4. MG Infection Downregulates MMP16 Expression

**Figure 4:**
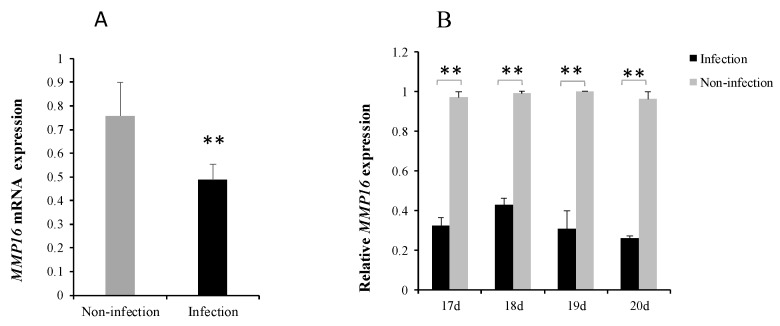
*MMP16* expression in DF-1 cells and the lungs of chicken embryos upon MG infection. The expression of *MMP16* in DF-1 cells (**A**) and in the lungs tissues on the 8 to 11 days post-infection (**B**) was measured by RT-qPCR and normalized to GAPDH. Uninfected lung tissues and DF-1 cells acted as negative controls. Values are mean ± SD, ** *p* < 0.01.

To further identify the role of *MMP16* in *MG*-HS infection, quantitative polymerase chain reaction (qRT-PCR) was adopted to measure *MMP16* expression. It was significant that *MMP16* expression level was lower in the infection group (Figure 4A). Eight to eleven days after infection (amount 17 to 20 days of egg hatching), *MMP16* expression was significantly decreased in MG-infected lungs of chicken embryos (Figure 4B). These results further demonstrate that *MMP16* expression is repressed within limits by gga-miR-146c in vivo and in vitro.

### 3.5. gga-miR-146c Is of Importance in NF-κB Pathway Regulation

**Figure 5:**
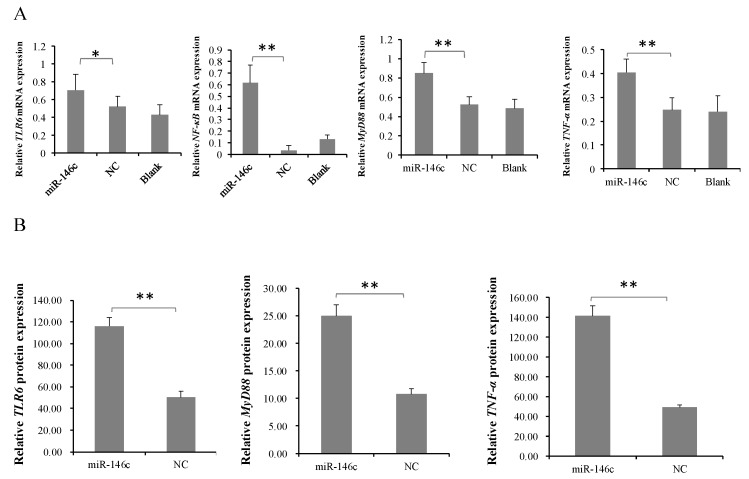

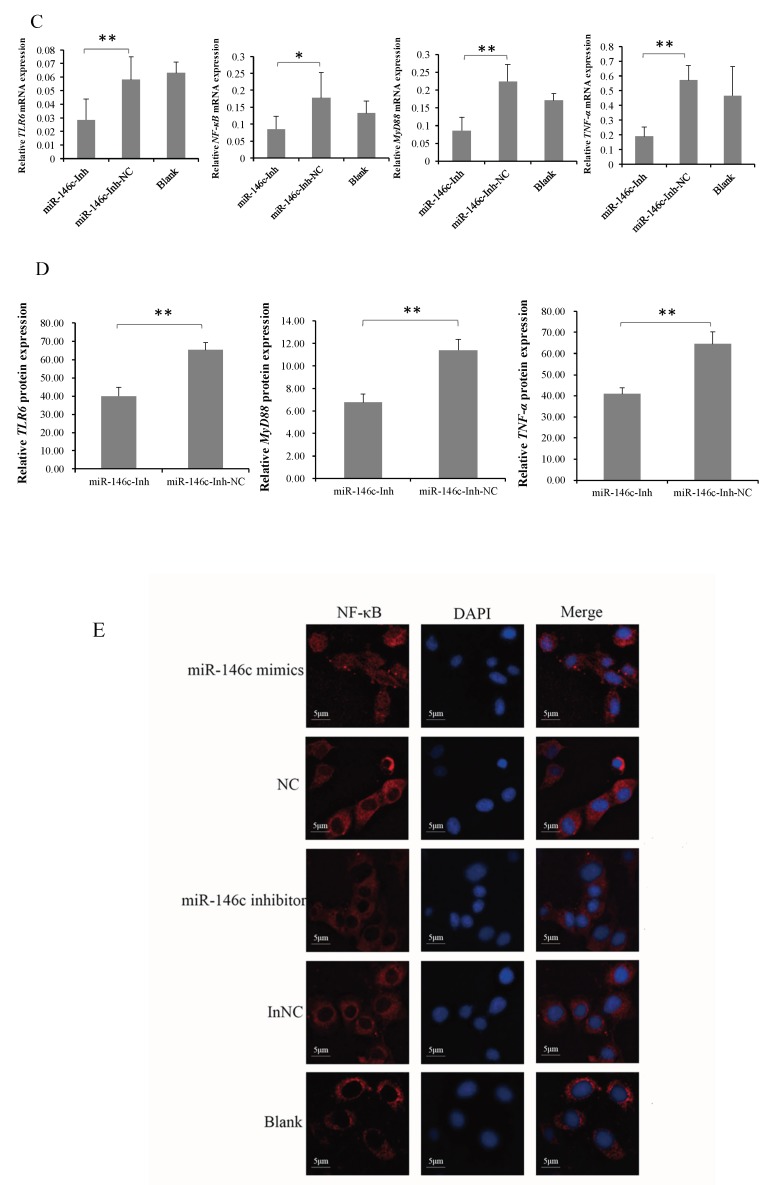
gga-miR-146c regulates activity of TLR6, NF-κB p65, MyD88 and TNF-α. Overexpression of gga-miR-146c activates TLR6, NF-κB p65, MyD88, and TNF-α after 48 h transfection. (**A**) Expressions of TLR6, NF-κB p65, MyD88, and TNF-α were measured via RT-qPCR; (**B**) Protein expressions of TLR6, MyD88, and TNF-α wer measured through ELISA. Inhibition of gga-miR-146c reduced TLR6, NF-κB p65, MyD88, and TNF-α expressions in DF-1 cells 48 h post-transfection; (**C**,**D**) The mRNA and protein expressions of TLR6, NF-κB p65, MyD88, and TNF-α were measured through RT-qPCR or ELISA, respectively, normalized to GAPDH; Values are mean ± SD, * *p* < 0.05, ** *p* < 0.01; (**E**) DF-1 cells were respectively transfected with gga-miR-146c, negative control, miR-146c-Inh or miR-146c-Inh-NC.The mock transfection served as blank. Stained macrophages were magnified 200×. gga-miR-146c overexpression made NF-κB p65 (red) turning into the cell nucleus.

Our previous study suggests that IL2/IL6-mediated inflammatory responses are induced with *MG* infection via TLR6/MyD88/NF-κB pathway [36]. To identify whether gga-miR-146c activates the TLR6/MyD88/NF-κB pathway to defend against *MG* infection, gga-miR-146c, miR-146c-NC, and miR-146c-Inh or miR-146c-Inh-NC were transfected into DF-1 cells for 48 h. The qRT-PCR results revealed that it is the gga-miR-146c expression, but not miR-146c-NC, significantly increased TLR6, NF-κB p65, MyD88, and TNF-α expression levels (Figure 5A), whereas miR-146c inhibitor significantly decreased the expression of these four genes (Figure 5C). The results of immunofluorescence showed NF-κB p65 protein was in the nucleus of DF-1 cells which transfected with gga-miR-146c, whereas the other groups had no clear change (Figure 5E). The proteins of TLR6, MyD88, and TNF-α in the cell supernatant treated with gga-miR-146c were higher than the cells treated with miR-146c-NC (Figure 5B), whereas the miR-146c inhibitor significantly decreased the proteins expression of TLR6, MyD88, and TNF-α (Figure 5D), and qRT-PCR results have the same trend with the ELISA results. These data indicate that gga-miR-146c inhibit *MMP16* expression within limits, and then activate the NF-κB signaling pathway in inflammation response.

### 3.6. Upregulation of gga-miR-146c Promotes Proliferation and Cycle Progression by Repressing Apoptosis

**Figure 6:**
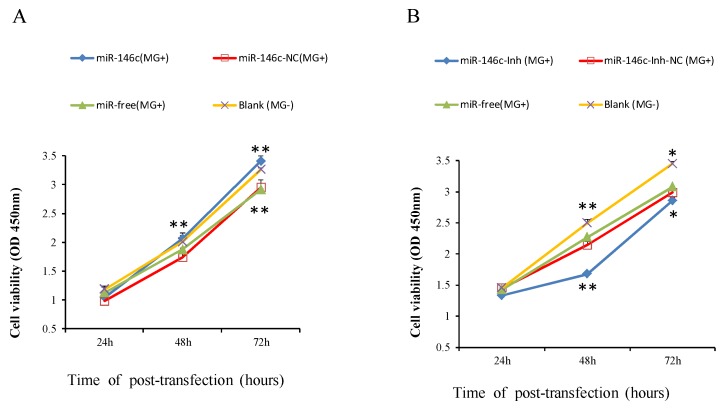
Proliferation of DF-1 cells was regulated by gga-miR-146c. Four groups of transfection treatments were carried out. Values are mean ± SD, * *p* < 0.05, ** *p* < 0.01. (**A**) Cell multiplication was dramatically promoted with gga-miR-146c overexpressed; (**B**) Cell multiplication was restrained by gga-miR-146c inhibitor.

To explore biological functions of gga-miR-146c in *MG*-HS infection, we focused on influences of gga-miR-146c on cell proliferation, apoptosis, and cycle. Four groups of transfection treatments were carried out. Cells transfected with gga-miR-146c mimics before MG infection were marked as miR-146c (*MG*+); cells transfected with miR-146c-NC were marked as miR-146c-NC (*MG*+); non-transfected cells were marked as miR-free (*MG*+). All above groups were infected with *MG*. Uninfected DF-1 cells were marked as blank (*MG*-). Cell Counting Kit-8 was adopted to calculate the results. It is showed that overexpressed miR-146c (*MG*+) group and the blank (*MG*-) group significantly increased the cell multiplication at 48 h and 72 h post-transfection compared to miR-146c-NC (*MG*+) group or miR-free (*MG*+) group (Figure 6A). At the same time, miR-146c inhibitor was assayed for further validation on cell proliferation. As expected, it led to significant reduction in cell proliferation after 48 h and 72 h transfection compared to each other three groups. A significant reduction of cell proliferation was also observed in miR-146c-Inh-NC (*MG*+) or miR-free (*MG*+) group (Figure 6B).

**Figure 7:**
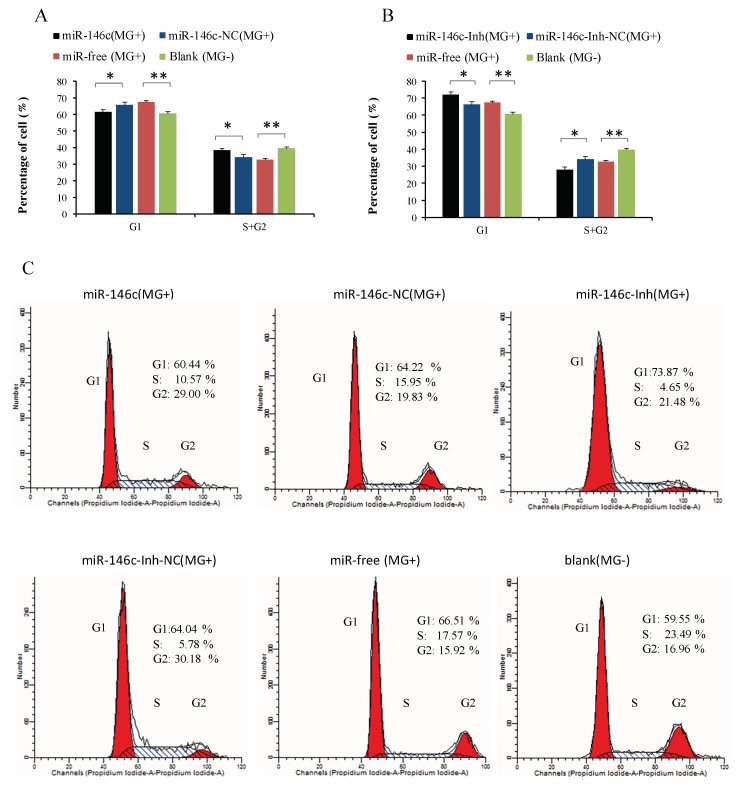
Cycle procession of DF-1 cells was affected by gga-miR-146c. Four groups of transfection treatments were carried out. Values are mean ± SD, * *p* < 0.05, ** *p* < 0.01. (**A**,**C**) Cell cycle was dramatically promoted with gga-miR-146c overexpression; (**B**,**C**) Cell cycle was suppressed by gga-miR-146c inhibitor.

To explore how gga-miR-146c promotes cell proliferation, the cell cycle and apoptosis of DF-1 cells were assayed by a flow cytometer. Similarly, DF-1 cells were transfected with the synthetic RNA oligonucleotides. *MG* infection disturbed mitosis by guiding cells arrested in the G1 cell cycle. The number of cells that stayed in S and G2 phases increased remarkably due to gga-miR-146c overexpression (Figure 7A). On the contrary, miR-146c-Inh led to an opposite result (Figure 7B).

**Figure 8:**
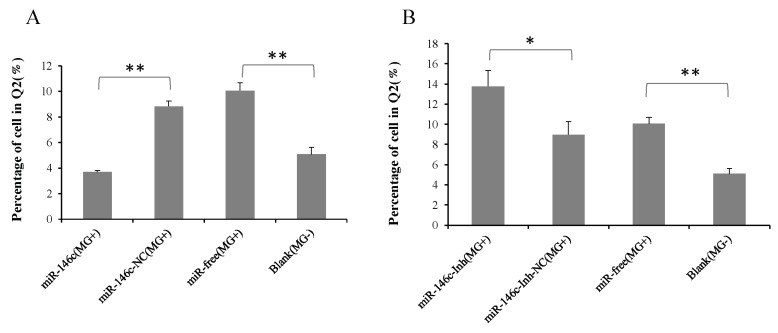

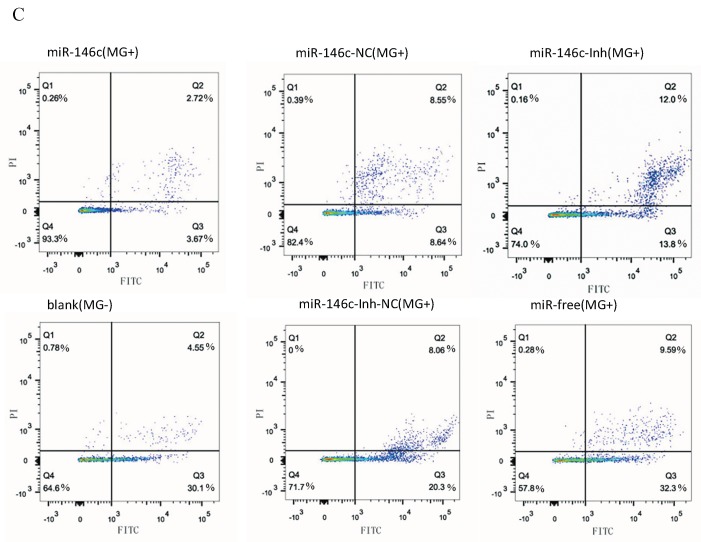
Cell apoptosis was influenced by gga-miR-146c. Four groups of transfection treatments were carried out. Values are mean ± SD, * *p* < 0.05, ** *p* < 0.01. (**A**,**C**) Cell apoptosis was significantly suppressed by gga-miR-146c overexpression; (**B**,**C**) Cell apoptosis was promoted with gga-miR-146c inhibitor.

According to cell apoptosis analysis results, gga-miR-146c overexpression effectively decreased apoptosis rates compared to the miR-146c-NC (*MG*+) group or the miR-free group (*MG*+) (Figure 8A). Opposite results were obtained when cells were treated with a miR-146c inhibitor (Figure 8B).

The above results indicate that upregulation of gga-miR-146c by *MG*-HS significantly enhances DF-1 cells proliferation upon *MG* infection by inhibiting cell apoptosis and enhancing cell cycle progression to defend against host *MG* infection.

## 4. Discussion

*MG* causes severe inflammation in the tracheas and lungs of chickens [37]. Recently, it was reported that numerous miRNAs play a pivotal role in the pathogenesis of inflammatory and avian diseases [38,39]. For example, gga-let-7i, gga-let-7b, gga-miR-221, and gga-miR-222 may participate in tumorigenesis induced by avian leucosis virus J [40,41]. gga-miR-130a suppresses Marek’s disease lymphoma cell multiplication and migration [42]. The gga-miR-199-3p, gga-miR-140-3p, and gga-miR-221-5p were repressed in Marek’s disease lymphoma [43]. Previously, we reported the roles of gga-miR-19a, gga-miR-99a, and gga-miR-101-3p in *MG*-HS infection of chickens [17,18,19]. However, the role of miRNAs in *MG*-HS infection of chickens is largely unknown.

The miR-146 family has important regulatory functions in many diseases, including influenza, virus infection, and cancer [44]. gga-miR-146c was upregulated in chicken embryos lungs upon *MG* infection according to deep sequencing data [31]. In this study, we demonstrated that gga-miR-146c was remarkably upregulated in both cells (Figure 1A) and lungs (Figure 1B). It is further indicated that *MMP16* is the target gene of gga-miR-146c (Figure 2D and Figure 3). *MMP16* expression was significantly decreased in MG-infected DF-1 cells and chicken embryos (Figure 4). These results indicate that miR-146c might participate in *MG* infection by binding to 3′-UTR region of *MMP16*. Meanwhile, *MG* infection is a complex process, so there may be other factors regulating *MMP16* expression. It remains to be further studied.

The significant upregulation of gga-miR-146c by *MG* prompted us to investigate the biological function of gga-miR-146c in DF-1 cell line. MMPs, a family of zinc-dependent endopeptidases, are of importance in pathological processes, including inflammation, pulmonary diseases, cardiovascular diseases, and cancer. [45,46]. MMP9 promotes Hepatitis B virus replication [47], and is of significant importance in response to gonococcal infection [48]. In melanoma, *MMP16* mediates a proteolytic switch that promotes cell adhesion and lymphatic invasion [49]. MiR-146b-5p targets *MMP16* to dramatically inhibit glioma and pancreatic cancer migration and invasion [50]. MiR-146a downregulated the expression of *MMP16* in caco-2 cells [51]. MiR-145 suppresses osteosarcoma metastasis via targeting *MMP16* [52].

NF-κB, a major transcription factor related to inflammatory signaling, is primarily involved in regulation of response to inflammatory and pathogens [53]. Bacteria challenged with *Mycoplasma pneumoniae* can release classical proinflammatory cytokines [54], which was shown to active NF-κB [55]. It is well-known that many microRNAs participate in the NF-κB pathway [56]. For example, the NF-κB pathway is activated due to the loss of miR-514a-3p in human testicular germ cell tumors [57]. The upregulation of miR-155 and miR-21 suppress the NF-κB pathway in HCV- or HIV-infected cells [58]. MiR-17-92 promotes leukemogenesis and activation of the NF-κB signaling [45]. Upregulated miR-429 inhibits the migration of HCC cells via the NF-κB pathway [59]. MiR-191 represses angiogenesis by the NF-κB pathway activation [60]. The NF-κB pathway was drastically ameliorated when MMP3 was decreased [61]. Multiple pathogens ligands, including bacteria, viruses, fungi, and protozoa, can be recognized by Toll-like receptors (TLRs) [62,63]. TLRs activate NF-κB-mediated inflammatory responses when stimulated by carcinogenic microbes and endogenous molecules [64]. NF-κB was found to mainly interact with TLRs [65]. The NF-κB pathway was inactivated for TLR6 knockout [66]. In our previous study, TLR6 were upregulated upon *MG* infection, followed by the upregulation of downstream NF-κB-mediated inflammatory responses. After TLR6 knockdown, DF-1 cells cannot make inflammatory response to *MG* infection via TLR6/NF-κB pathway [36]. In this study, the overexpression of gga-miR-146c led to a remarkable increase the expression of TLR6, NF-κB p65, TNF-α, and MyD88 in DF-1 cells (Figure 5A,B,E), whereas gga-miR-146c inhibitor extremely repressed their expression (Figure 5C–E). gga-miR-146c may affect downstream genes TLR6 and MyD88 to active TLR6/MyD88/NF-κB pathway through its target genes. TLR6 signaling pathway is activated to produce TNF-α which is one of inducible factors of NF-κB. We demonstrate that *MMP16* is a direct target gene of gga-miR-146c, while it is still possible that other target genes of gga-miR-146c contribute to the observation. Recently, we reported that expression of NF-κB, TNF-α, and MyD88 was increased in chicken embryonic lungs upon *MG* infection as well as in DF-1 cells [17]. Together, it is rational to believe that upregulation of gga-miR-146c is involved in pathogenesis of *MG* infection to activate TLR6/MyD88/NF-κB pathway.

The NF-κB signaling pathway is closely associated with DF-1 cells proliferation [67]. The miR-146a could promote cell proliferation by suppressing activation of the NF-κB signaling pathway [68,69,70]. The miR-146a could suppress cell cycle progression in non-small cell lung cancer [71]. Downregulation of miR-146b promotes the proliferation rate of fusion cells [72]. Inhibition of NF-κB significantly suppressed MMP9 gene expression [73]. The metastatic thyroid cancer was related with NF-κB/MMP9 high expression [74]. MiR-132 affects glima cell migration and invasion by *MMP16* [75]. Silence of *MMP16* expression significantly decreased the invasion and proliferation capacity of gastric cancer (GC) [76]. Our results indicate that *MG* infection inhibits DF-1 cells multiplication by blocking the transition from the G1 phase to S and G2 phases, and upregulated miR-146c significantly promotes cell proliferation by inhibiting cell apoptosis and enhancing cell cycle progression (Figure 6A, Figure 7A, and Figure 8A), whereas the miR-146c inhibitor restrained cell proliferation by promoting cell apoptosis and inhibiting cell cycle progression (Figure 6B, Figure 7B, and Figure 8B). As we can see, *MG* infection and gga-miR-146c have opposite effect on cell proliferation and apoptosis. In the miR-free (*MG*+) group, cells were infected with *MG* only. DF-1 cells were damaged upon *MG* infection. Then, gga-miR-146c was upregulated to react to *MG* infection. In the miR-146c (*MG*+) group, gga-miR-146c mimics was transfected to DF-1 cells firstly, and after 4 h, cells were infected with *MG*. Therefore, gga-miR-146c in miR-146c (*MG*+) group worked earlier and more effective compared with miR-free (*MG*+) group. In general, gga-miR-146c promotes proliferation, inhibits cell apoptosis, and finally, prevents *MG* infection. In conclusion, gga-miR-146c upregulation upon *MG* infection inhibits *MMP16* expression within limits, in turn activating TLR6/MyD88/NF-κB pathway to promote the cell proliferation and the cell cycle progression by decreasing cell apoptosis to defend against *MG* infection. As both gga-miR-146c and *MMP16* are highly conserved in multiple species (Figure 2C), we propose that there may be a similar strategy for the other species to defend against *Mycoplasma* infection. However, further studies are needed to support this prediction.

## Supplementary Materials

The following are available online at https://www.mdpi.com/2073-4409/8/5/501/s1, Tables S1 and S2.
