# Supplementary material for: gga-miR-146c Activates TLR6/MyD88/NF-κB Pathway through Targeting MMP16 to Prevent Mycoplasma Gallisepticum (HS Strain) Infection in Chickens
Source: Cells. 2019 May 24;8(5):501. doi: 10.3390/cells8050501 (PMC6562429; doi:10.3390/cells8050501)
Supplement: Supplementary file 1 [file cells-08-00501-s001.pdf]

Article

gga-miR-146c Activates TLR6/MyD88/NF-κB Pathway through Targeting MMP16 to Prevent *Mycoplasma Gallisepticum* (HS Strain) Infection in Chickens

Kang Zhang †, Yun Han †, Zaiwei Wang, Yabo Zhao, Yali Fu and Xiuli Peng *

Key Laboratory of Agricultural Animal Genetics, Breeding and Reproduction Ministry of Education, College of Animal science and Technology and College of Veterinary Medicine, Huazhong Agricultural University, Wuhan 430070, China; zhangkang123@webmail.hzau.edu.cn (K.Z.); hany@webmail.hzau.edu.cn (Y.H.); wangzaiwei@webmail.hzau.edu.cn (Z.W.); zyb@webmail.hzau.edu.cn (Y.Z.); FYL@webmail.hzau.edu.cn (Y.F.)

* Correspondence: xlpengsishun@mail.hzau.edu.cn; Tel: +86-27-87281396; Fax: +86-27-87280408

† These authors contributed equally to this work.

Table 1. Sequences of DNA primers.

Name	Primer sequence (5'-3')	Accession No.
Primers for 3'-UTR Cloning		
MMP16 3'-UTR-F	TGCCTCGAGACCTTCTGGTTTTCCC	XM-015277626
MMP16 3'-UTR-R	TTGGCGGCCGCACTCCCCTCAAATTC	XM-015277626
Mut-MMP16 3′-UTR-F	TAGCTGCCCAAGATATGGCTATTTTTTTG TTTCTATTCTTGTTTTTAAAAATAAAGC	
Mut-*MMP16* 3'-UTR-R	GAATAGAAACAAAAAAATAGCCATATC TTGGGCAGCTAATGCAGTCTCA	
Primers for RT-qPCR		
GAPDH-F	GAGGGTAGTGAAGGCTGCTG	NM-204305
GAPDH-R	CACAACACGGTTGCTGTATC	NM-204305
RT- gga-miR-146c	CTCAACTGGTGTCGTGGAGTCGGCAATT CAGTTGAGCAGTCCAT	MIMAT-0007735
gga-miR-146c-F	CTGGTCGGTGAGAACTGAAT	MIMAT-0007735
gga-miR-146c-R	CAACTGGTGTCGTGGAGTCGGC	MIMAT-0007735
gga-5s-rRNA-F	CCATACCACCCTGGAAACGC	
gga-5s-rRNA-R	TACTAACCGAGCCCGACCCT	
MMP16 -F	TGGACCAACAGACCGAGAC	XM-015277626
MMP16 -R	CCAAGACAAGGAGGCACAA	XM-015277626
MyD88-F	TCAGTTTGTCCAGGAGATG	NM-001030962
MyD88-R	GGTGTAATGAACCGCAAGATA	NM-001030962
NF-κB -F	GCCAGGTTGCCATCGTGT	NM-205129
NF-κB -R	CGTGCGTTTGCGCTTCTC	NM-205129
TNF-α-F	GGACAGCCTATGCCAACAAG	XM-015294124
TNF-α-R	ACACGACAGCCAAGTCAACG	XM-015294124

Table 2. Sequences of RNA oligonucleotides.

Name	Sequences (5'-3')
gga-miR-146c mimics	UGAGAACUGAAUUCCAUGGACUG<br>GUCCAUGGAAUUCAGUUCUCAUU
gga-miR-146c NC sense	UUCUCCGAACGUGUCACGUTT
gga-miR-146c NC antisense	ACGUGACACGUUCGGAGAATT
gga-miR-146c inhibitor	CAGUCCAUGGAAUUCAGUUCUCA
gga-miR-146c inhibitor NC	CAGUACUUUUGUGUAGUACAA
